# Phenotypic Diversity of Breast Cancer-Related Mutations in Metalloproteinase-Disintegrin ADAM12

**DOI:** 10.1371/journal.pone.0092536

**Published:** 2014-03-20

**Authors:** Yue Qi, Sara Duhachek-Muggy, Hui Li, Anna Zolkiewska

**Affiliations:** Department of Biochemistry and Molecular Biophysics, Kansas State University, Manhattan, Kansas, United States of America; Institute of Molecular and Cell Biology, Biopolis, United States of America

## Abstract

Six different somatic missense mutations in the human *ADAM12* gene have been identified so far in breast cancer. Five of these mutations involve highly conserved residues in the extracellular domain of the transmembrane ADAM12-L protein. Two of these extracellular mutations, D301H and G479E, have been previously characterized in the context of mouse ADAM12. Three other mutations, T596A, R612Q, and G668A, have been reported more recently, and their effects on ADAM12-L protein structure/function are not known. Here, we show that ADAM12-L bearing the G668A mutation is largely retained in the endoplasmic reticulum in its nascent, full-length form, with an intact N-terminal pro-domain. The T596A and R612Q mutants are efficiently trafficked to the cell surface and proteolytically processed to remove their pro-domains. However, the T596A mutant shows decreased catalytic activity at the cell surface, while the R612Q mutant is fully active and comparable to the wild-type ADAM12-L. The D301H and G479E mutants, consistent with the corresponding D299H and G477E mutants of mouse ADAM12 described earlier, are not proteolytically processed and do not exhibit catalytic activity at the cell surface. Among all six breast cancer-associated mutations in ADAM12-L, mutations that preserve the activity - R612Q and L792F - occur in triple-negative breast cancers, while loss-of-function mutations - D301H, G479E, T596A, and G668A - are found in non-triple negative cancers. This apparent association between the catalytic activity of the mutants and the type of breast cancer supports a previously postulated role of an active ADAM12-L in the triple negative breast cancer disease.

## Introduction

Disintegrin and metalloproteinase domain-containing protein ADAM12 is a member of the ADAM family of proteins that mediate cleavage of substrates at the cell surface and/or modulate intracellular signaling pathways [1], [2]. ADAM12 is highly up-regulated in human breast tumors [3]–[9]. In triple negative breast cancers (TNBCs, lacking estrogen receptor and progesterone receptor expression and *ERBB2* gene amplification), high expression of *ADAM12-L,* but not *ADAM12-S,* mRNA is associated with poor prognosis [10]. *ADAM12-L* and *ADAM12-S* are two different splice variants that encode the long, transmembrane protein isoform ADAM12-L and the short, secreted ADAM12-S, respectively [11].

Among thirteen different *ADAM* genes that encode catalytically active proteases [1], *ADAM12* is the most frequently somatically mutated gene in human breast cancers. As of September 2013, the COSMIC database (Catalogue of Somatic Mutations in Cancer, http://cancer.sanger.ac.uk/cancergenome/projects/cosmic/) listed 6 confirmed somatic missense mutations in the *ADAM12* gene per a total of 1104 unique breast carcinoma samples analyzed. The frequencies of breast cancer-associated missense mutations in other genes encoding catalytically active *ADAMs* were: 1/973 in *ADAM9*, 1/973 in *ADAM10*, 1/1147 in *ADAM17*, 1/1010 in *ADAM19*, 1/973 (plus one nonsense mutation) in *ADAM20*, and 1/973 in *ADAM30*. No other missense/nonsense somatic mutations were reported for the remaining *ADAM* genes encoding catalytically active proteases (i.e., *ADAM8, −15, −21, −28, −33*, and *ADAMDEC1*). A relatively high frequency of mutations in the *ADAM12* gene can be attributed to the fact that *ADAM12* is located on human chromosome 10q26.2, in a region capable of forming highly stable secondary structures [12].

**Figure 1:**
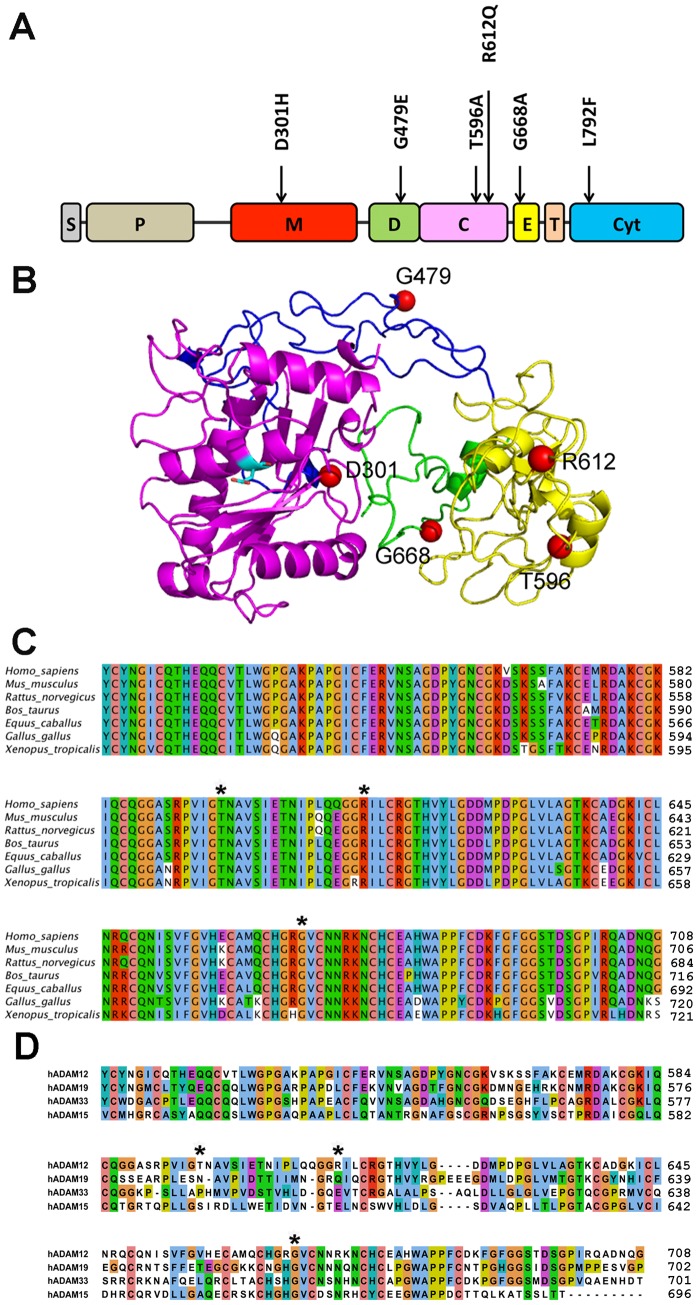
Breast cancer-associated mutations in human ADAM12-L. (A) A diagram of human ADAM12-L. Six non-synonymous mutations identified in human breast cancers are indicated. S, signal peptide; P, prodomain; M, metalloproteinase domain; D, disintegrin domain; C, cysteine-rich domain; E, EGF-like domain; T, transmembrane region; Cyt, cytoplasmic tail. (B) Model of the extracellular domain of human ADAM12-L generated by the I-TASSER protein structure prediction tool (C-score *−*0.26, estimated TM accuracy of the model 0.68±0.12 [20], [21]). The metalloproteinase, disintegrin, cysteine-rich, and EGF-like domains are shown in purple, blue, yellow, and green, respectively. Positions of the five amino acids mutated in breast cancers (red spheres) and the side chain of the catalytic residue E351 (cyan sticks) are indicated. (C) Sequence alignment of the cysteine-rich and EGF-like domains of ADAM12 from different species. NCBI RefSeq numbers are: *Homo_sapiens*, NP_003465; *Mus_musculus*, NP_031426; *Rattus_norvegicus*, XP_001054670; *Bos_taurus*, NP_001001156, *Equus_caballus*, XP_001490097; *Gallus_gallus*, NP_001136322, and *Xenopus_tropicalis*, NP_001035103. (D) Sequence alignment of the cysteine-rich and EGF-like domains of human ADAM12 and the most closely related human ADAMs. NCBI RefSeq numbers are: ADAM19, XP_005266060, ADAM33, NP_079496.1, and ADAM15, NP_997080. In C and D, asterisks indicate three novel mutations in human ADAM12 found in breast tumors [14], [15]. Clustal X color scheme was applied.

The six breast cancer-associated mutations in the ADAM12-L protein include the D301H mutation in the metalloproteinase domain, G479E in the disintegrin domain, T596A and R612Q in the cysteine-rich domain, G668A in the epidermal growth factor (EGF)-like domain, and L792F in the cytoplasmic tail [13]–[15] (Figure 1A). We have previously shown that the D299H and G477E mutations in mouse ADAM12 (which correspond to the D301H and G479E mutations in human ADAM12) are loss-of-function mutations that inhibit the intracellular trafficking and proteolytic activation of the nascent ADAM12 protein [16]. The L792F mutation in human ADAM12-L was reported not to affect protein processing, localization, or function [16], [17]. The other three mutations - T596A, R612Q, and G668A - have been identified more recently [14], [15], and their effects on the structure/function of ADAM12 are currently unknown.

Here, we show that there is functional diversity between the three recently identified mutations. While ADAM12-L containing the G668A mutation is largely retained in the endoplasmic reticulum (ER) and is not proteolytically activated in the Golgi, the T596A mutant is properly trafficked and proteolytically processed but is still catalytically inactive. The R612Q mutant is trafficked, processed, active, and is indistinguishable from the wild-type (WT) ADAM12-L. Taking into consideration all six known breast cancer-associated somatic mutations in ADAM12-L, we note an apparent association between the catalytic activity of ADAM12-L mutants and the molecular characteristic of the tumor. The two mutations that do not have any effect on ADAM12-L activity, R612Q and L792F, occurred in TNBCs. The four mutations that render ADAM12-L inactive, i.e., D301H, G479E, G668A, and T596A, were described in non-TNBCs. This observation further suggests an important role of the catalytically active ADAM12-L in the triple negative breast cancer disease.

## Materials and Methods

### Expression Constructs

Retroviral expression vector *ADAM12-L*-pBABEpuro was used for the expression of the wild-type (WT) ADAM12-L protein. The D301H, G479E, T596A, R612Q and G668A point mutations were introduced by site-directed mutagenesis using the QuickChange kit (Stratagene). The entire lengths of all DNA inserts were sequenced to confirm that no other mutations were introduced during mutagenesis. The expression construct of Delta-like 1 (DLL1) in pIRESpuro vector was described earlier [18].

### Cell Culture and Treatment

Human MCF10A mammary epithelial cells (ATCC) were cultured in DMEM/F12 (1∶1) supplemented with 5% horse serum, 0.5 μg/ml hydrocortisone, 20 ng/ml human EGF, 10 μg/ml insulin, 100 ng/ml cholera toxin, and 1% penicillin/streptomycin. Retrovirus production and stable transduction of MCF10A cells with viruses encoding WT or mutant ADAM12-L, or with control viruses bearing empty pBABEpuro vector, were performed as described previously [19]. Transient transfections were performed using X-tremeGENE HP transfection reagent (Roche). For protein stability assay, cells were treated with 10 μg/ml cycloheximide (EMD Millipore) in culture medium for indicated times.

### Antibodies

Anti-ADAM12 rabbit polyclonal antibody (Ab 3394) specific for the cytoplasmic domain of human ADAM12-L was developed in our laboratory, as described [10]. The remaining antibodies were: anti-ADAM12 mouse mAb (R&D Systems; clone 632525, specific for the extracellular domain of ADAM12-L), anti-KDEL mouse mAb (clone 10C3, Enzo Life Sciences), anti-EGFR (D38B1) XP rabbit mAb (Cell Signaling), anti-phospho-EGFR (pY1173) rabbit polyclonal Ab (R&D Systems), anti-DLL1 rabbit polyclonal Ab (H-265, Santa Cruz Biotechnology), anti-β-actin mouse mAb (clone AC-15, Sigma), and anti-α-tubulin mouse mAb (clone DM 1A, Sigma).

### Immunofluorescence

Stably transduced MCF10A cells were plated on glass coverslips placed in 6-well plates. Two days later, cells were fixed with 3.7% paraformaldehyde/DPBS for 20 min, followed by permeabilization with 0.1% Triton X-100/DPBS for 5 min. Coverslips were incubated with anti-ADAM12-L polyclonal antibody (1∶500 dilution) and anti-KDEL antibody (1∶200 dilution), followed by incubation with rhodamine Red-X-conjugated anti-rabbit IgG antibody, Alexa 488-conjugated anti-mouse IgG antibody, and DAPI. Immunofluorescence was examined using an Axiovert 200 inverted fluorescent microscope.

### Flow Cytometry

Cells were trypsinized into a single cell suspension, washed with DPBS containing 3% BSA, and incubated with anti-ADAM12 monoclonal antibody or isotype control antibody (R&D Systems, both at 1∶10 dilution) for 30 minutes on ice. Cells were then washed 3 times, incubated with allophycocyanin (APC)-conjugated anti-mouse antibodies (Jackson ImmunoResearch; 1∶100) for 30 min on ice, washed again, and then incubated with 1 μg/ml propidium iodide (PI; BD Biosciences) for viability. Analysis was performed using a BD FACSCalibur flow cytometer. Only the cells negative for PI staining (viable cells) were selected for the ADAM12 analysis.

### Cell Surface Biotinylation and Western Blotting

Cells were washed with DPBS, incubated for 60 min at 4°C with 2.5 mM EZ-link NHS-PEG_12_-biotin (Thermo Scientific), and then washed with ice-cold 100 mM glycine/DPBS. Cellular proteins were extracted with extraction buffer (50 mM Tris-HCl, pH 7.4, 150 mM NaCl, 1% Triton X-100, 1% sodium deoxycholate, 0.1% SDS, 1 mM 4-(2-aminoethyl)-benzene-sulfonylfluoride hydrochloride (AEBSF), 5 μg/ml aprotinin, 5 μg/ml leupeptin, 5 μg/ml pepstatin A, 10 mM 1,10-phenanthroline; 0.5 ml buffer/well in a 6-well plate). Cell extracts were centrifuged at 21,000×g for 15 min at 4°C, and supernatants were incubated for 1 h at 4°C with NeutrAvidin sepharose (GE Healthcare; 0.5 ml cell extract/25 μl of resin). The resin was washed three times with extraction buffer, eluted with SDS sample buffer; samples were then resolved by SDS-PAGE and transferred to a nitrocellulose membrane. Western blotting was performed using anti-ADAM12 polyclonal (1∶20,000 dilution), anti-DLL1 (1∶1,000), anti-EGFR (1∶5,000 dilution), anti-pY1173 EGFR (1∶5,000) primary antibodies and HRP-conjugated secondary antibodies, as described [10]. Signal detection was performed using WestPico chemiluminescence detection kit (Pierce).

### Endo H Treatment

Stably transduced cells were treated with extraction buffer and centrifuged at 21,000×g for 15 min at 4°C. Supernatants were treated with EndoH_f_ denaturing buffer (New England BioLabs), boiled, and then treated with Endo H_f_ (3,000 U), according to the manufacturer’s instructions.

### Determination of Cell Doubling Times

Stably transduced cells were seeded in 6-well plates at the density of 60,000 cells/well. After 24, 48, and 72 h, cells were detached and counted with Cellometer AutoT4 (Nexcelom Bioscience), in duplicates. Exponential growth curves were fitted to each dataset and the doubling times were calculated using a nonlinear regression function in the GraphPad Prism 5.0 software.

### Evaluation of ADAM12-L-mediated Shedding of EGFR Ligands

MCF10A cells stably overexpressing WT or mutant ADAM12-L proteins, or cells stably transduced with empty pBABEpuro vector, were incubated for 16 h in serum-free media. Conditioned media were then collected, pre-cleared by centrifugation, and added to the duplicate wells containing “reporter” empty vector-transduced MCF10A cells that were pre-incubated for 16 h in serum-free medium. After 30 min, cells were washed with DPBS, treated with extraction buffer containing phosphatase inhibitors 50 mM NaF, 2 mM Na_3_VO_4_, and 10 mM Na_4_P_2_O_7_, and analyzed by SDS-PAGE and Western blotting using anti-phospho-EGFR (pY1173) and anti-EGFR antibodies.

### Cell Migration Assay

MCF10A cells with stable overexpression of WT or mutant ADAM12-L proteins, or control empty vector-transduced cells, were suspended in MCF10A medium containing 0.1% BSA instead of horse serum, and seeded in the upper chambers of Transwell inserts with a 8-μm pore size polyethylene terephthalate membrane (BD Biosciences), at 2.5×10^4^ cells/chamber. The lower chambers contained the full culture medium supplemented with 5% horse serum and 20 ng/ml EGF. After incubation at 37°C for 18 h, cells were fixed with 3% glutaraldehyde in DPBS for 20 min, washed twice with DPBS, and stained with 0.5% crystal violet in 20% methanol for 10 min. Cells at the upper face of the membrane were removed with cotton swabs, and cells at the lower face were examined with an inverted microscope using a 10× magnification, and photographed. Numbers of cells in five random fields were counted, and the mean number of migrated cells for each insert was determined.

### ADAM12-L Structure Prediction

The I-TASSER software (http://zhanglab.ccmb.med.umich.edu/I-TASSER/, refs. [20], [21]) was used to predict the structure of the extracellular domain of the mature form of human ADAM12-L (amino acids 208–708). The ADAM22 template (PDB:3G5C) was excluded from the I-TASSER template library due to a compact packing of the metalloproteinase domain against the cysteine-rich domain and steric hindrance of the pseudo-catalytic site [22], a feature that may be characteristic for catalytically inactive ADAMs.

### Statistical Analysis

Fisher’s exact test, unpaired *t* test, and linear regression were performed using GraphPad Prism 5.0.

## Results

The five breast cancer-associated mutations mapping to the extracellular portion of ADAM12-L are located in different domains and spread over a region spanning more than 300 amino acids (Figure 1A). We asked whether these mutations might be clustered in a particular region in the three-dimensional structure of the protein. Since the X-ray structure of human ADAM12-L is not available, we used the I-TASSER protein structure prediction tool [20], [21] to build a 3D model of the extracellular domain of the active form of human ADAM12-L comprising the metalloproteinase, disintegrin, cysteine-rich, and EGF-like domains. From the model, it is apparent that the cancer-associated mutations are scattered over the entire structure and they do not cluster in a particular region of the protein (Figure 1B).

**Figure 2:**
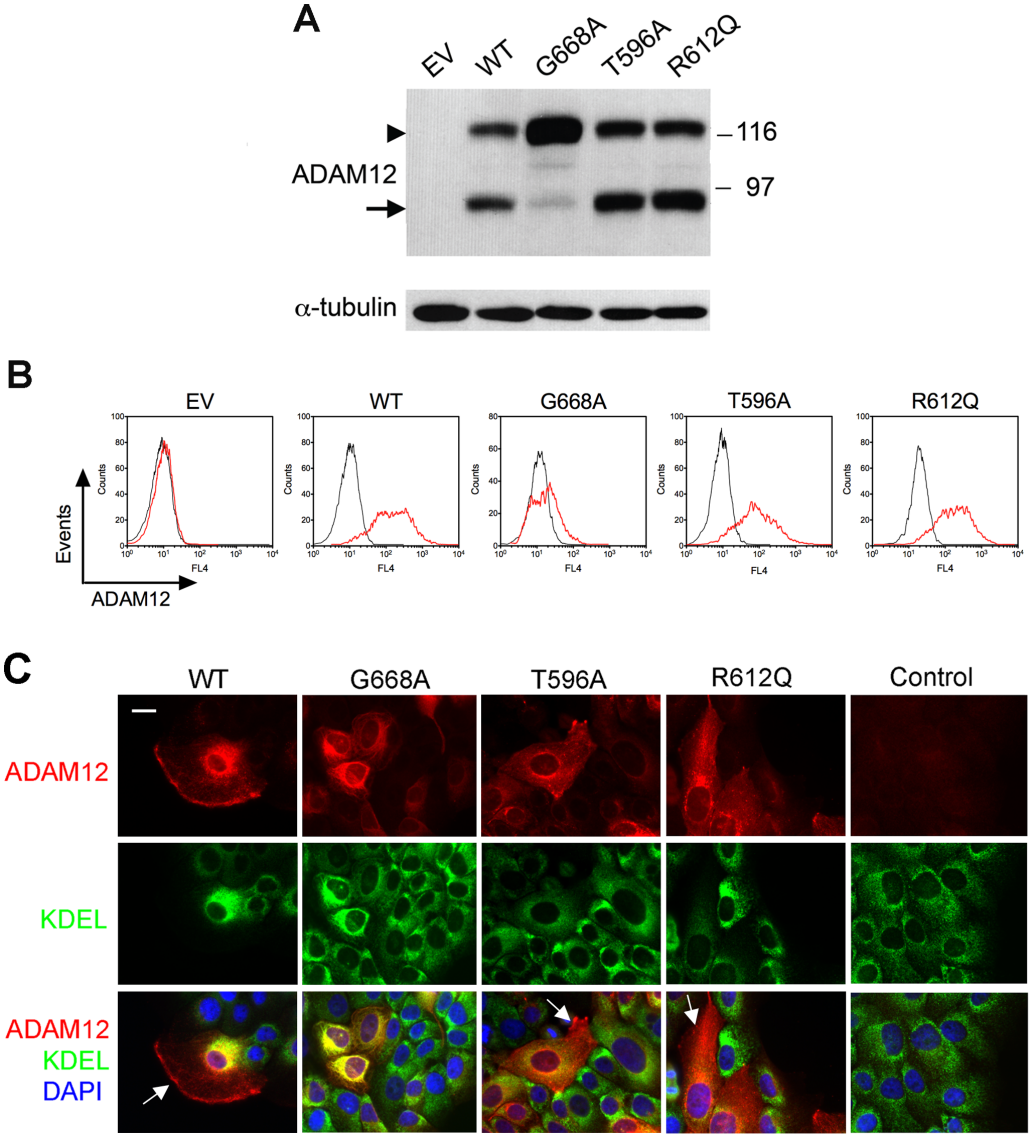
The effect of G668A, T596A, and R612Q mutations on the proteolytic processing and intracellular localization of ADAM12-L. (A) Proteolytic processing of the WT and mutant forms of human ADAM12-L in MCF10A cells. Cells with stable expression of ADAM12-L proteins or control empty vector (EV)-transduced cells were selected with puromycin after retroviral infection. Total cell lysates were analyzed by Western blotting using antibody specific for the cytoplasmic tail of ADAM12-L. Arrowhead indicates the nascent, full-length, catalytically inactive form, and arrow denotes the mature, processed, catalytically active form of ADAM12-L. (B) Cell surface localization of ADAM12-L was examined by flow cytometry. Live cells were trypsinized and stained with an antibody specific for the extracellular domain of ADAM12-L (red) or with isotype control antibody (black). (C) Intracellular localization of the WT and mutant ADAM12-L proteins. Cells were co-stained with anti-ADAM12 antibody (red), anti-KDEL antibody (endoplasmic reticulum marker; green), and DAPI (blue). Control represents cells expressing WT ADAM12-L, incubated with pre-immune serum instead of anti-ADAM12 antibody. Arrows indicate ADAM12 staining in post-ER compartments. Bar, 20 μm.

The three recently identified mutations - T596A, G668A, and R612Q - involve amino acid residues in ADAM12 that are highly conserved between species (Figure 1C). The G668 residue is also conserved between human ADAM12 and the most closely related human ADAMs, namely ADAM19, ADAM33, and ADAM15 [1]. In contrast, the T596 and R612 residues are not conserved between ADAM12-L and the other three ADAMs (Figure 1D). To study the effects of the T596A, G668A, and R612Q mutations on the functionality of ADAM12-L, we stably expressed WT and mutated ADAM12-L proteins in human MCF10A mammary epithelial cells. Western blotting of total cell lysates with an antibody specific for the cytoplasmic tail of ADAM12-L demonstrated that the proteolytic processing of WT ADAM12-L and the T596A and R612Q mutants was virtually indistinguishable (Figure 2A). In all three cases, the mature, processed form of ∼90-kDa was easily detected, and its abundance was similar to that of the nascent, full-length form of ∼120-kDa. In contrast, processing of the G668A mutant was significantly inhibited. Staining of live cells with an antibody recognizing the extracellular domain of ADAM12-L and analysis by flow cytometry demonstrated that WT ADAM12-L and the T596A and R612Q mutants were readily detected at the cell surface, whereas the G668A mutant showed much weaker cell surface staining (Figure 2B). To further explore the intracellular localization of WT ADAM12-L and the mutants, we performed immunofluorescence staining of permeabilized cells with anti-ADAM12 and anti-KDEL antibody, a marker of the ER. We observed that substantial amounts of WT ADAM12-L and the T596A and R612Q mutants were present in post-ER compartments located at the cell periphery (Figure 2C), consistent with cell surface localization of these proteins detected by flow cytometry. In contrast, anti-ADAM12 staining in G668A-expressing cells largely coincided with anti-KDEL staining (Figure 2C), suggesting that this mutant was retained, at least partially, in the ER and explaining why it was poorly detected at the cell surface (Figure 2B).

**Figure 3:**
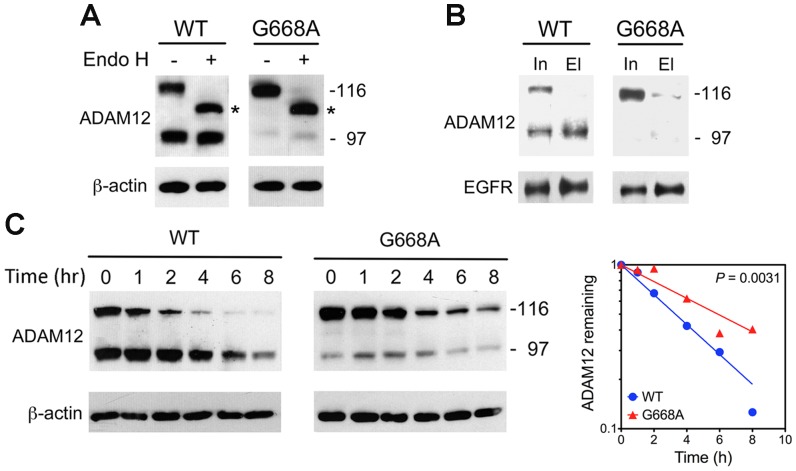
The G668A mutation causes retention of ADAM12-L in the endoplasmic reticulum. (A) Probing the maturation of ADAM12-L proteins in post-ER compartments by treatment with endoglycosidase H (Endo H). Total cell lysates were incubated for 1 h with Endo H, followed by Western blotting with anti-ADAM12 antibody. Asterisk indicates the full-length, de-glycosylated form of ADAM12-L. (B) Cell surface biotinylation of ADAM12-L proteins. Intact cells were incubated for 1 h with membrane-impermeable NHS-PEG_12_-biotin, followed by isolation of biotinylated proteins using NeutrAvidin beads and Western blotting with anti-ADAM12 antibody. Input (In) refers to total cell lysates prior to Neutravidin binding, and eluate (El) refers to biotinylated proteins that bound to the resin. Biotinylation of epidermal growth factor receptor (EGFR) served as positive control. (C) Protein stability assay. Cells were incubated with 10 μg/ml of cycloheximide for the indicated times, followed by immunoblotting. Band intensities of ADAM12-L (nascent and mature forms combined) were quantified by densitometry, normalized to β-actin, and analyzed using a single exponential decay model. Half-lives of the WT and the G668A mutant ADAM12-L were significantly different (3.3 h *vs* 5.9 h, respectively, *P* = 0.0031).

The pronounced effect of the G668A mutation on the processing and localization of ADAM12-L is unexpected, given the very conservative nature of the Gly-to-Ala substitution. Thus, the maturation of the G668A mutant in the secretory pathway was further probed with endoglycosidase H (Endo H) and compared to the WT ADAM12-L. When the lysate of WT-expressing cells was treated with Endo H, the mobility of the nascent ADAM12-L increased, while the mature form was more resistant to the Endo H treatment (Figure 3A). This result indicated that the mature form, but not the nascent form, progressed through the Golgi compartment, where the resistance of N-linked oligosaccharides to Endo H is acquired. The G668A mutant, represented predominantly by the full-length form, was sensitive to Endo H, further suggesting that this mutant did not efficiently progress beyond the ER. Finally, we performed cell surface biotinylation of intact cells expressing the WT or G668A mutant protein. In WT ADAM12-L-expressing cells, the mature form was efficiently biotinylated, whereas the nascent form was resistant to the modification (Figure 3B). This result indicated that at the cell surface of WT ADAM12-L-expressing cells, the mature ADAM12-L form was much more abundant than the nascent form. In G668A mutant-expressing cells, the extent of cell surface biotinylation of ADAM12-L was considerably lower than in WT ADAM12-expressing cells, further indicating that the G668A mutation impaired trafficking of the ADAM12-L protein to the cell surface.

The next question might be: Why is the G668A mutant inefficiently trafficked to the cell surface and predominantly retained in the ER? We reasoned that this mutant might be misfolded and retained by the protein quality control system operating in the ER. However, using co-immunoprecipitation, we did not detect interaction between the G668A mutant (or WT ADAM12-L) with ER chaperones BiP, Grp94, and calnexin, or with ER stress proteins that assist in proper disulfide formation ERp44, ERp57, ERp72, Ero1, PDI (results not shown). Also, cellular levels of these chaperones/stress proteins were not elevated in G668A-expressing cells. Cycloheximide chase experiments further demonstrated that the G668A mutant was in fact more stable than the WT ADAM12-L protein. Estimated half-life of the G668A mutant was 5.9 h, which was significantly larger than the half-life of WT ADAM12-L (3.3 h, Figure 3C). Collectively, these results suggested that although the G668A mutant was retained in the ER, most likely it was not misfolded and it was not subject to rapid degradation.

Next, we asked about the catalytic activity of ADAM12-L mutants. While a mutation causing impaired intracellular trafficking of ADAM12-L is naturally expected to cause a decrease in the enzyme activity at the cell surface (unless it exerts an indirect effect, such as enhancing cell surface expression of a different proteolytic enzyme), a mutation that does not affect intracellular trafficking and processing can still have an impact on the catalysis or substrate recognition. For comparison, we also included the D301H and G479E mutants in the current analysis. The corresponding D299H and G477E mutations in mouse ADAM12 were shown previously to block the intracellular trafficking and processing of the protein, as well as ADAM12-mediated cleavage of the substrate protein DLL1 [16]. Trafficking, processing, and catalytic activities of human D301H or G479E mutants have not been examined before. The L792F mutation in the cytoplasmic tail of human ADAM12-L was reported to have no effect on ADAM12-L trafficking, processing, or catalytic activity [16], [17].

**Figure 4:**
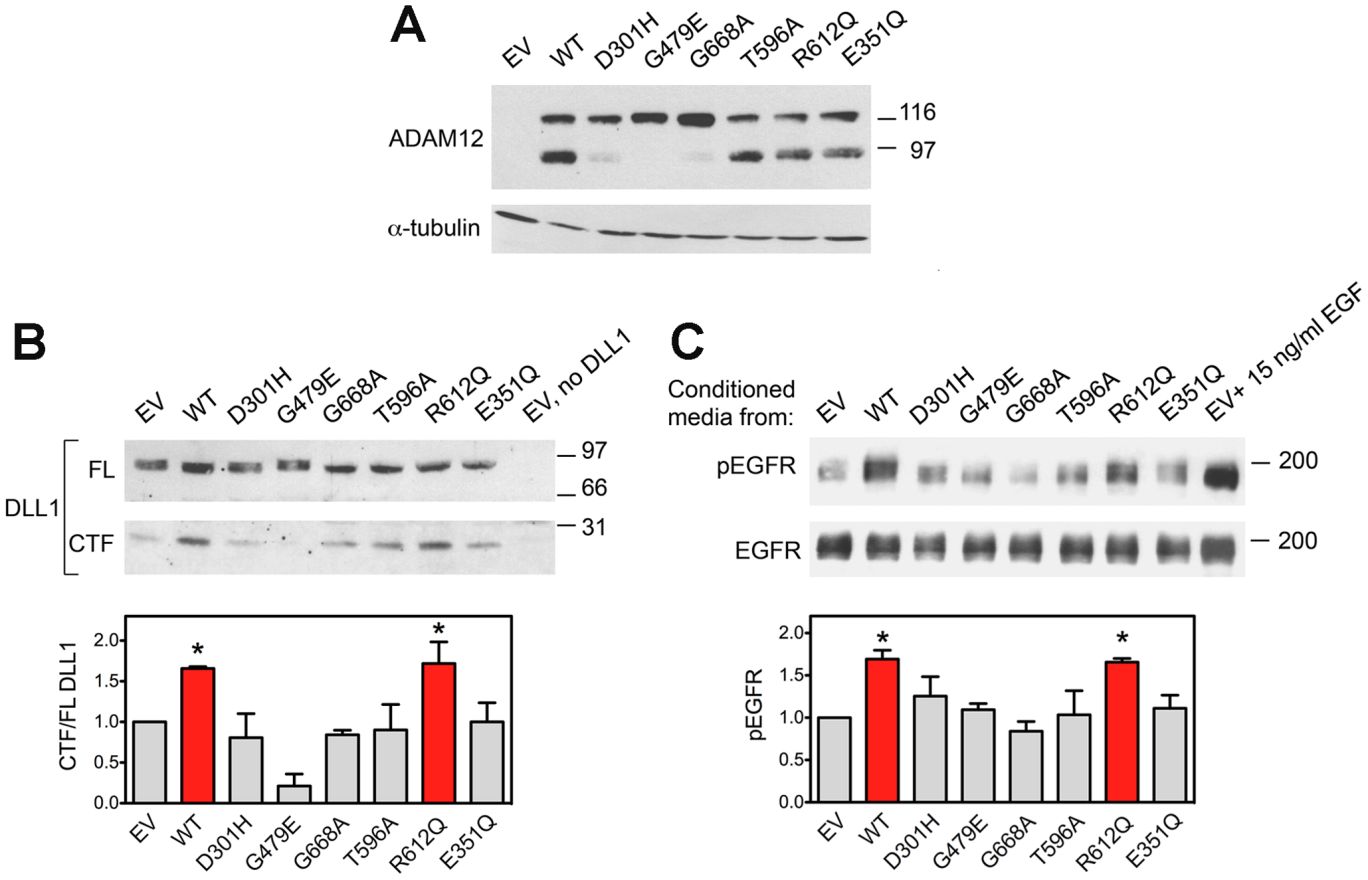
Cell-based assays of the catalytic activity of ADAM12-L mutants. (A) MCF10A cells with stable overexpression of WT or mutant ADAM12-L proteins, or control empty vector (EV)-transduced cells used in assays shown in panels B and C. The E351Q mutant is catalytically inactive due to the mutation at the active site and is used as negative control. (B) Cells shown in panel A were transiently transfected to express a substrate protein Delta-like 1 (DLL1). Cell extracts were subjected to Western blotting with anti-DLL1 antibody. The full-length (FL) DLL1 and the C-terminal fragment (CTF) generated by the proteolytic cleavage are indicated. Band intensities were quantified by densitometry. The experiment was repeated two times, mean values ± SEM are shown. *, *P*<0.05. Notice that the amount of the C-terminal fragment (CTF) of DLL1 is increased in WT- and R612Q mutant ADAM12-L-expressing cells. (C) Cells shown in panel A were serum-starved for 16 h. Conditioned media were then transferred to reporter MCF10A cells that were also pre-starved for 16 h, and incubation continued for 30 min. The level of phosphorylation of EGFR in reporter cells was evaluated by Western blotting using anti-phosho-Y1173 antibody, band intensities were quantified by densitometry, and the extent of phosphorylation of EGFR (pEGFR) normalized to the total EGFR protein was evaluated. Conditioned media from EV-transduced cells supplemented with 15 ng/ml EGF served as positive control. The experiment was repeated three times, mean values ± SEM are shown. *, *P*<0.05. Notice that EGFR phosphorylation in reporter cells was increased upon adding conditioned media from WT- or R612Q mutant ADAM12-L-expressing cells.

We used three different approaches to evaluate the catalytic activity of the D301H, G479E, T596A, R612Q, and G668A mutants at the surface of MCF10A cells (Figure 4A). In the first approach, cells with stable overexpression of WT or mutant ADAM12-L were transfected with a plasmid encoding DLL1. The full-length (FL) DLL1 and the C-terminal fragment (CTF) generated by the proteolytic cleavage were detected by Western blotting, as described previously [16], [18]. We observed that the CTF/FL ratio of DLL1 was higher in cells expressing WT ADAM12-L or the R612Q mutant than in control cells, indicative of the catalytic activity of these ADAM12 proteins toward the DLL1 substrate (Figure 4B). In contrast, the CTF/FL ratio was not increased in D301H, G479E, G668A, or T596A mutant-expressing cells compared to control cells, suggesting that these four mutations significantly reduced the ability of ADAM12-L to cleave DLL1.

In the second approach, we focused on ADAM12-L-mediated shedding of EGFR ligands, as this function of ADAM12-L has been recently shown to be important in the biology of TNBC [10]. Among different EGFR ligands, ADAM12-L was previously shown to cleave EGF [23], [24] and heparin-binding(HB)-EGF [25], [26]. Here, the amount of endogenous EGFR ligands shed to the media by MCF10A cells stably overexpressing WT or mutant ADAM12-L proteins was evaluated. Cells were incubated for 16 h in serum-free media, the conditioned media were then transferred to starved “reporter” MCF10A cells, incubated for 30 min, and then the extent of EGFR phosphorylation at Tyr1173, one of the major autophosphorylation sites in response to ligand binding [27], was examined. Conditioned media from WT ADAM12-L or R612Q mutant-expressing cells increased EGFR phosphorylation in reporter cells (Figure 4C). In contrast, conditioned media from D301H, G479E, G668A, or T596A mutant-expressing cells did not cause elevation in EGFR phosphorylation (Figure 4C), suggesting that these mutants most likely did not efficiently shed EGFR ligands.

**Figure 5:**
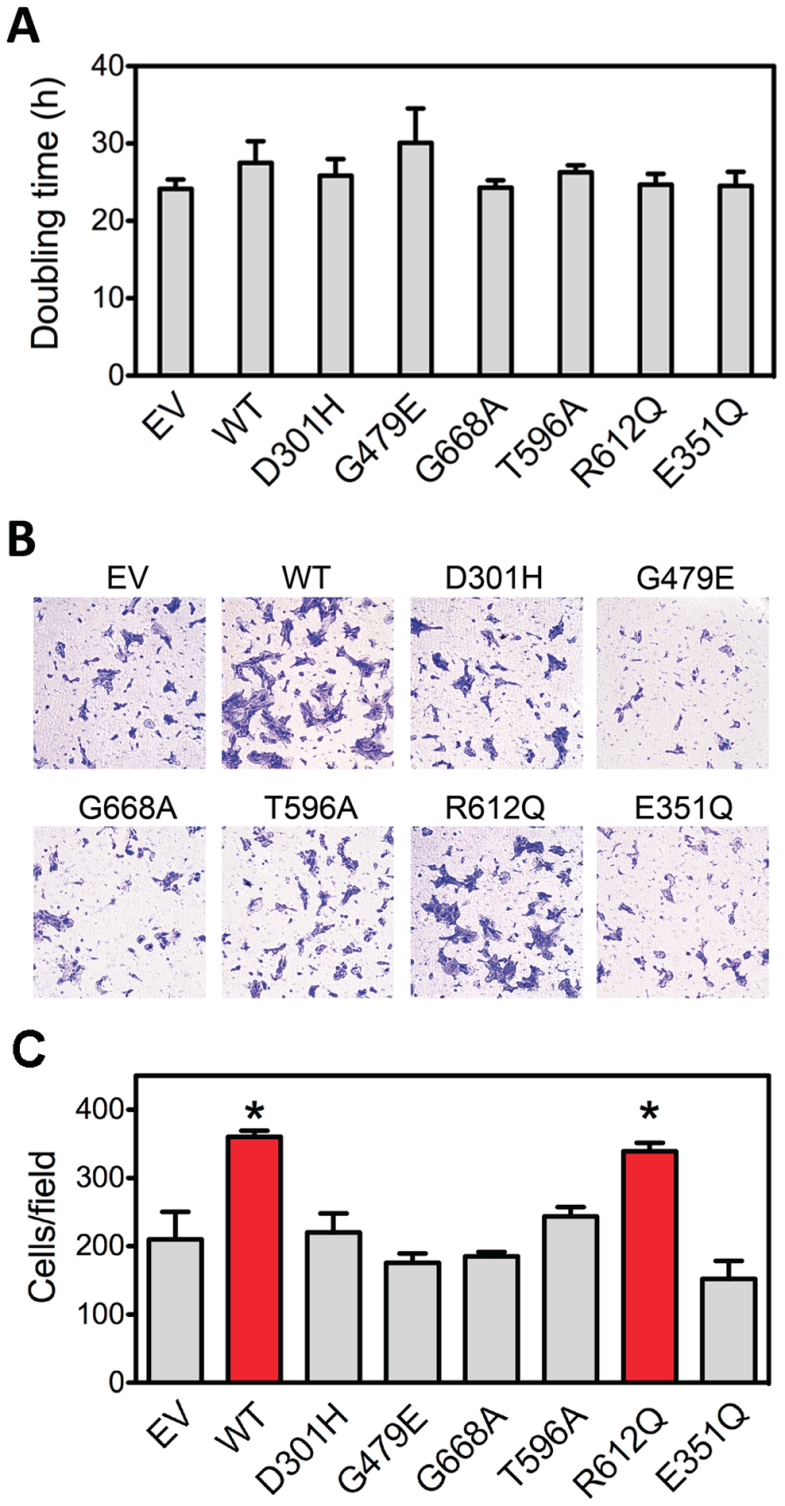
The effect of WT and mutant ADAM12-L on cell growth and migration. (A) Doubling times of MCF10A cells with stable overexpression of WT or mutant ADAM12-L proteins, or control empty vector (EV)-transduced cells. The data are shown as means with 95% confidence intervals based on 3 independent determinations. (B) Cells were analyzed for migration using Transwell assays. Representative images of crystal violet-stained cells present at the lower face of the Transwell inserts are shown. (C) Quantification of the migration assay. The numbers of migrated cells were counted in five random fields for each insert. The data are shown as mean ± SEM from 3 determinations. *, *P*<0.05.

In the third approach, we investigated the effect of WT and mutant ADAM12-L expression on cell migration. We showed previously that overexpression of mouse ADAM12 in NIH3T3 cells increased cell migration using scratch wound assay, and that the D299H and G477E mouse ADAM12 mutants were inactive [16]. ADAM12-L has been also found to potentiate the migration of head and neck squamous cell carcinoma cells [28]. However, overexpression of ADAM12-L in breast cancer MCF-7 cells did not affect cell migration [29], and mouse ADAM12 was reported to inhibit keratinocyte migration or integrin α4β1-mediated CHO cell migration cells [30], [31]. Thus, the effect of ADAM12 on cell migration appears to be highly context-dependent and may involve distinct mechanisms. In the current study, we used a Transwell assay to assess the effect of mutations in human ADAM12-L on the migration of MCF10A cells. While overexpression of WT ADAM12-L or the mutants did not affect cell growth (Figure 5A), the WT and the R612Q mutant ADAM12-L significantly increased cell migration (Figure 5B and C). This up-regulation of cell migration required the catalytic activity of ADAM12-L, because the E351Q mutant-expressing cells migrated at a rate similar to control cells. Importantly, overexpression of the D301H, G479E, G668A, or T596A mutants did not increase cell migration, further indicating that these mutants were either not efficiently targeted to the cell surface or had activities significantly lower than the WT ADAM12-L and the R612Q mutant.

A summary of the functional characterization of breast-cancer associated ADAM12-L mutations, as well as the properties of breast tumors in which each mutation was identified, is provided in Table 1. Included in Table 1 are also SIFT scores and PolyPhen scores obtained from the Ensembl Genome Browser (www.ensembl.org). SIFT is a sequence homology-based tool that sorts intolerant from tolerant amino acid substitutions and predicts whether an amino acid substitution in a protein will have a phenotypic effect (http://sift.jcvi.org, ref. [32]). PolyPhen-2 is a tool which predicts the variation effect on protein function based on physical and comparative considerations (http://genetics.bwh.harvard.edu/pph2; ref. [33]). SIFT scores ≤0.05 and PolyPhen scores ≥0.95 designate amino acid substitutions that are predicted to be damaging to the structure/function of a protein. As summarized in Table 1, with the exception of the T596A mutation, there is an agreement between the predicted effect of each mutation and the experimentally determined activity of ADAM12-L. Importantly, mutations that do not have impact on the ADAM12-L activity - R612Q and L792F - were found in TNBCs. Mutations that inhibit the ADAM12-L activity - D301H, G479E, G668A, and T596A - occurred in non-TNBCs. Thus, there is an apparent association between the intact activity of ADAM12-L and the triple-negative status of tumors, although this association is only borderline significant due to the small sample size (*P* = 0.0667, Fisher’s exact test).

**Table 1:** Summary of breast cancer-associated somatic mutations in ADAM12-L.

Mutation	Molecular characteristics	Sample source	Zygosity	SIFT^a^	PolyPhen^b^	ER retention	Activity	Reference for ER retention/Activity
D301H	non-TNBC	Cultured	Heterozygous	**0**	**1**	++	−	[16] and this study
G479E	non-TNBC	Tumor sample	Heterozygous	**0**	**1**	+++	−	[16] and this study
G668A	non-TNBC	Tumor sample	Unknown	**0.001**	**0.998**	++	−	This study
T596A	non-TNBC	Tumor sample	Unknown	0.09	**0.999**	−	−	This study
R612Q	TNBC	Tumor sample	Unknown	0.28	0.89	−	+	This study
L792F	TNBC	Cultured	Heterozygous	0.35	0.223	−	+	[17]

a Predicted effect of each mutation on ADAM12-L function according to the SIFT algorithm [32]. SIFT scores ≤0.05 indicate amino acid substitutions that are predicted to be damaging (shown in bold).

b Predicted effect of each mutation on ADAM12-L function according to the PolyPhen-2 tool [33]. Scores represent prediction confidence that a given mutation changes the protein function; scores ≥0.95 are indicated in bold.

## Discussion

The current study, together with two other previous reports [16], [17], provides an insight into the structural/functional aspects of the currently known breast cancer-associated mutations in ADAM12. These mutations are scattered along the entire length of the protein, and they do not appear to cluster within a specific region in the three-dimensional model of ADAM12-L. Among three novel mutations characterized for the first time in this study, only one - G668A - has a severe impact on the intracellular trafficking and proteolytic processing of the protein. Considering the very conservative nature of the Gly-to-Ala substitution, this finding is somewhat unexpected. However, as Gly is highly flexible and can adopt conformations that are forbidden for other amino acids, it is possible that the replacing Gly with Ala at position 668 is not compatible with the native structure of ADAM12-L. High conservation of Gly668 between ADAM12-L and other related ADAMs (Figure 1D) further suggests the importance of a Gly residue at this position.

The G668A mutation adds to the other two mutations that were previously found to cause ER retention and a lack of proteolytic processing of mouse ADAM12, and are now confirmed to inhibit the processing of human ADAM12-L, i.e., D301H and G479E. While we do not find any evidence that these mutants are unfolded and rapidly degraded, they must assume a significantly different conformation from the WT ADAM12-L to be retained in the ER. Clearly, since these three mutants exist mostly as the ∼120-kDa precursors, with the inhibitory pro-domain intact, they are expected to be catalytically inactive. The lack of catalytic activity of the D301H, G479E, and G668A mutants has been confirmed here by the DLL1 cleavage assay, by the EGFR activation assay, and by the Transwell migration assay.

The effects of D301H, G479E, and G668A mutations on the structure/function of ADAM12-L agree well with the effects predicted by the SIFT and PolyPhen tools (see Table 1). Interestingly, the T596A mutation is predicted to be tolerated by SIFT, but harmful by the PolyPhen-2 algorithm. We find that the T596A substitution does not affect intracellular processing of ADAM12, and this result is consistent with the fact that with Thr596 is poorly conserved between ADAM12-L and other closely related human ADAMs (Figure 1D). However, we find that the T596A mutation renders ADAM12-L inactive at the cell surface. Thr596 is not located at the active site and most likely it is positioned distantly from the metalloproteinase domain in the three-dimensional structure of ADAM12 (Figure 1B). It is currently unclear whether the T596A substitution exerts a long-range inhibitory effect on the catalytic site or whether it blocks the interaction of ADAM12-L with its substrates. Overall, we conclude that the D301H, G479E, T596A, and G668A mutations should be classified as loss-of-function mutations.

The most striking observation emerging from this study is the apparent association between the catalytic activity of ADAM12-L mutants and the type of breast cancer. Two mutations that did not have any impact on the catalytic activity of ADAM12-L, R612Q and L792F, were found in triple-negative tumors. In contrast, all four loss-of-function mutations, D301H, G479E, T596A, and G668A, occurred in non-triple-negative tumors. Thus, it appears that loss-of-function mutations in ADAM12-L tend to be excluded from TNBCs. However, a larger patient population and possibly a broader mutation spectrum are needed to test whether there is indeed a significant association between the type of ADAM12-L mutations and the triple-negative status of breast tumors. Interestingly, the *ADAM12* gene is located in the genomic region that has been recently found to be significantly deleted in Luminal B tumors (cytoband 10q26.11, wide peak boundaries chr10∶104674916-135534747, q value 0.0056373) [34]. Low q-values associated with gene amplifications/deletions (typically below 0.25) suggest that amplifications/deletions at a particular locus are enriched by selective pressures [35]. The same study found that the *ADAM12* gene was hypermethylated and showed lower expression in Luminal B tumors than in other types of breast cancer [35]. As Luminal B tumors are estrogen receptor-positive [36], [37], these results, together with our functional analysis of ADAM12 breast cancer-related mutants presented here, collectively suggest that ADAM12 may play fundamentally different roles in TNBCs and in non-TNBC.

We have recently postulated that ADAM12-L may be the primary protease responsible for the activation of EGFR in early stage, lymph node-negative TNBCs [10]. This conclusion was supported by decreased distant metastasis-free survival times of patients with high expression levels of *ADAM12-L*, increased EGFR phosphorylation in a mouse xenograft model of breast cancer, and a strong correlation between the level of anti-ADAM12-L and anti-phospho-EGFR immunostaining in human breast tumor samples. We have also noticed a positive correlation between *ADAM12-L* and *HB-EGF* and *EGFR* in TNBCs, but not in receptor-negative non-TNBCs [10]. In estrogen receptor-positive MCF-7 breast cancer cells, overexpression of ADAM12-L promoted estrogen-independent proliferation, and this effect of ADAM12-L was linked to elevated EGFR activation [9]. Furthermore, a recent study demonstrated cancer cells under hypoxia up-regulate ADAM12-L expression, leading to increased HB-EGF shedding, EGFR activation, formation of invadopodia, and cancer invasion [26]. We believe that these results, together with the analysis of breast cancer-associated ADAM12-L mutants described here, point to an important role of ADAM12-L in the pathology of triple negative breast cancer.
